# Safety and clinical outcomes of computed tomography‐guided percutaneous microwave ablation in patients aged 80 years and older with early‐stage non‐small cell lung cancer: A multicenter retrospective study

**DOI:** 10.1111/1759-7714.13209

**Published:** 2019-11-03

**Authors:** Xiaoying Han, Xia Yang, Guanghui Huang, Chunhai Li, Licheng Zhang, Yuanxun Qiao, Chuntang Wang, Yuting Dong, Xiangming Chen, Qingliang Feng, Chuandai Wang, Zhenhua Rong, Kun Ding, Zhigang Wei, Yang Ni, Jiao Wang, Wenhong Li, Min Meng, Xin Ye

**Affiliations:** ^1^ Department of Oncology Shandong Provincial Hospital Affiliated to Shandong University Jinan City China; ^2^ Shandong University Qilu Hospital Jinan China; ^3^ 960 Military Hospital of China Taian China; ^4^ Taian Hospital of Traditional Chinese Medicine Dezhou China; ^5^ The Second People Hospital of Dezhou Liaocheng China; ^6^ Dezhou People's Hospital Dezhou China; ^7^ Taishan Hospital affiliated to Taishan Medical College, Taian China; ^8^ Liaocheng Tumor Hospital, Liaocheng China; ^9^ Feicheng Hospital of Traditional Chinese Medicine Taian China; ^10^ The People's Hospital of Cao County Heze China; ^11^ Shouguang Hospital of Traditional Chinese Medicine Weifang China

**Keywords:** Microwave ablation, non‐small cell lung cancer, older patients

## Abstract

**Background:**

Previous studies have documented the therapeutic value of computed tomography (CT)‐guided percutaneous microwave ablation (MWA) for early‐stage non‐small cell lung cancer (NSCLC). However, few studies have focused on patients aged 80 years and older. This retrospective study aimed to evaluate the safety and clinical outcomes of CT‐guided percutaneous MWA in patients aged 80 years and older with early‐stage peripheral NSCLC.

**Methods:**

A retrospective analysis of 63 patients aged 80 years and older with cT1a‐2bN0M0 peripheral NSCLC who underwent CT‐guided percutaneous MWA was performed between January 2008 and January 2018 at 11 hospitals in Shandong Province, China.

**Results:**

The median follow‐up time was 21.0 months. The overall median survival time was 50 months. The cancer‐specific median survival time was not reached in five years. The one‐, two‐, three‐, four‐, and five‐year overall survival rates were 97.1%, 92.6%, 63.4%, 54.4%, and 32.6%, respectively. The one‐, two‐, and three‐year cancer‐specific survival (CSS) rates were 97.9%, 97.9%, and 69.4%, respectively. The four‐ and five‐year CSS rates were not achieved. A total of 14 patients (22.2%) had local progression. The one‐, two‐, three‐, four‐, and five‐year local control rates were 88.8%, 78.8%, 70.3%, 63.9%, and 63.9%, respectively. The mortality rate was 0% within 30 days after the procedure. Major complications included pneumothorax requiring drainage (21.1%), pulmonary infection (4.2%), and pleural effusions requiring drainage (2.8%).

**Conclusions:**

CT‐guided percutaneous MWA is a safe and effective modality for treating patients aged 80 years and older with early‐stage peripheral NSCLC.

## Key points

**Significant findings of the study:** Microwave ablation is a safe and effective treatment for patients aged 80 years and older with early‐stage peripheral non‐small cell lung cancer.

**What this study adds:** As a result of our findings, more older patients with early‐stage peripheral non‐small cell lung cancer may be eligible for treatment and may thus have a better prognosis.

## Introduction

Primary lung cancer remains the leading cause of cancer‐related death among men and women.^1^ In China, lung cancer is the most common type of cancer affecting men aged >75 and women >60 years.^2^ The number of older patients diagnosed with early non‐small cell lung cancer (NSCLC) has significantly increased with the aging population and the increased use of lung cancer screening technology. Surgical resection remains the treatment of choice for stage I NSCLC, although surgical methods continue to evolve.^3^, ^4^ However, more than 30% of patients aged >65 years with early NSCLC cannot tolerate surgical treatment because of poor lung function or other serious comorbidities.^5^ Moreover, a retrospective study of patients with stage I NSCLC who underwent lobar or sublobar resection (involving >70 000 patients) demonstrated that age (>75 years) was an independent risk factor for 30‐day postoperative mortality, and the extent of the surgical treatment appeared to affect the 30‐day mortality rate in the elderly.^6^ In the last decade, several new treatments, including stereotactic radiotherapy, chemotherapy, and tyrosine kinase inhibitors, have been developed for patients with lung cancer who would gain limited benefit from traditional chemotherapy or radiotherapy. Several image‐guided percutaneous thermal ablation technologies (eg, radiofrequency ablation [RFA], microwave ablation [MWA], and cryoablation) have been developed as favorable therapeutic options for patients who are not candidates for surgery because of poor cardiopulmonary reserve, anatomic constraints that limit resection, failure of traditional therapies, or refusal to undergo surgical procedures.^7^, ^8^, ^9^ Compared with RFA, MWA generates greater temperatures and requires shorter treatment time.^10^ In MWA, energy is not distributed by means of an electric current, which increases the heating radius in the poor thermal conduction environment of the lung.^11^ Despite these benefits of MWA, the safety and clinical outcomes of MWA in geriatric patients with early‐stage NSCLC are not well understood. Thus, this study aimed to evaluate whether MWA is a safe and effective therapy for patients aged 80 years and older with inoperable peripheral stage T1a‐T2bN0M0 NSCLC.

## Methods

This retrospective study was approved by the Institutional Review Boards of each of the following 11 hospitals: Shandong Provincial Hospital Affiliated to Shandong University, Qilu Hospital of Shandong University, 960 Military Hospital of China, Taishan hospital affiliated to Taishan Medical College, Taian Hospital of Traditional Chinese Medicine, The People's Hospital of Cao County, Feicheng Hospital of Traditional Chinese Medicine, Shouguang Hospital of Traditional Chinese Medicine, Liaocheng Tumor Hospital, Dezhou People's Hospital, and Dezhou Tumor Hospital. The requirement for informed consent was waived, and the study was conducted according to the Declaration of Helsinki.

The clinical records of patients with lung tumors who underwent MWA as their initial treatment in one of the hospitals mentioned above were reviewed. Patients (i) with stage I and lymph node‐negative IIa pathologically confirmed NSCLC; (ii) aged ≥80 years; (iii) with peripheral tumors; (iv) without medical history of other malignant tumors; (v) who refused to undergo alternative surgeries and (vi) with an Eastern Cooperative Oncology Group performance status of 0–2, and 7, whose length of follow‐up was at least six months, were eligible.

### Preablation assessment and procedural technique

For accurate clinical staging, all patients were recommended to undergo a whole‐body positron‐emission tomography‐computed tomography (PET‐CT) examination. If PET‐CT was not possible (such as for economic reasons), then enhanced craniocerebral magnetic resonance imaging, enhanced thoracic and abdominal CT, and whole‐body bone scan were suggested. The clinical staging was based on the Union for International Cancer Control TNM, eighth edition.^12^ Before ablation, all patients were evaluated by a multidisciplinary group, including a radiation oncologist, thoracic surgeon, thoracic radiologist, and medical oncologist. Anticoagulation and antiplatelet medications were temporarily discontinued 5–7 days before the procedure. Prophylactic antibiotic agents were not routinely administered before or after ablation.

The machine used for imaging guidance and monitoring was either a GE LightSpeed 16 Slice CT (GE), a Siemens SOMATOM Sensation 64 CT (Siemens), or a NeuViz 16 Platinum CT (Neusoft). The MTC‐3C microwave ablation system (Vison‐China Medical Devices R&D Center, CFDA Certification No. 20153251978), ECO‐100A1 microwave ablation system (ECO Medical Instrument Co., Ltd. CFDA Certification No. 20173251268), or KY‐2450B microwave ablation system (CANYOU Medical Inc., CFDA Certification No. 20153251727) was used. The main frequency was set to 2450 GHz, and the output power was 0–100 W (continually adjustable). The microwave antenna had an effective length of 100–180 mm and an outside diameter of 15–18 G, with a 15 mm active tip. The microwave antenna also had a water circulation cooling system to reduce its surface temperature.

Local anesthetic agents along with pre‐emptive analgesic agents were administered to patients. Pre‐emptive analgesia consisted of 10 mg of morphine and 10 mg of diazepam administered 30 minutes before treatment and 50 mg of flurbiprofen axetil administered 15 minutes before MWA treatment. None of the patients in this study required general anesthesia. The ablation power of the microwave generator was set at 60–80 W, and the ablation time was set to 20 minutes. Once patients were satisfactorily anesthetized, the microwave ablation antenna was inserted into the bottom of the tumor using CT‐guidance. A single antenna was used for tumors ≤3.0 cm in diameter, and two antennas were used simultaneously for tumors >3.0 cm in diameter.

### Follow‐up and clinical outcome evaluation

Treatment of a tumor with ablation is considered a technical success when the tumor is treated according to the protocol and is determined, at the time of the procedure, to be entirely overlapped or encompassed by the ablation zone plus an ablative margin.^13^ All patients underwent noncontrast chest CT 24 hours after the ablation procedure to detect early‐onset asymptomatic complications and ground‐glass opacities surrounding the lesions. Contrast‐enhanced chest CT was performed monthly for the first three months after the procedure. Enhanced chest CT or PET‐CT was subsequently performed every three months. The efficacy of local tumor ablation was assessed according to the standards drafted by Ye *et al*.^14^ The development of asymmetric or nodular uptake of 18F‐fluorodeoxyglucose at the ablation site or enlargement of the ablated tumor after initial shrinkage was considered a local recurrence if technical success had been confirmed. Overall survival (OS) was defined as the period from the initial procedure to death or final follow‐up.

### Complications and side effects

Complications were assessed according to the standards drafted by the International Working Group on Imagine‐Guided Tumor Ablation in 2014.^13^ Major complications were defined as life‐threatening conditions that developed during or after ablation and resulted in substantial damage and dysfunction that required either short‐term or prolonged hospitalization. Minor complications were defined as self‐limiting complications without sequelae that required only a short hospital stay for observation or treatment.

### Statistical analysis

The data were analyzed using SPSS for Windows Version 19.0 (IBM, Chicago, IL). Survival curves were constructed using the Kaplan‐Meier method.

## Results

### Patient demographics and tumor characteristics

From January 2008 to January 2018, 63 older patients (40 men and 23 women; mean age ± standard deviation: 82.1 ± 3.2 years; range: 80–95 years) were enrolled in this study; subjects either refused to undergo surgery (*n* = 14) or had cancer that was deemed inoperable (*n* = 49). The three commonest comorbidities were hypertension, coronary artery heart disease, and chronic obstructive pulmonary disease (COPD) (Table 1). A total of 28 patients (44.4%) had more than two comorbidities. Before being treated with MWA, all patients underwent CT‐guided percutaneous lung biopsy for pathological confirmation of their malignancies. Pathologies included adenocarcinoma, squamous cell carcinoma, and large cell lung cancer (Table 1). Because two patients were diagnosed with stage I adenocarcinoma with simultaneous occurrence in both lungs after pathological and PET‐CT scans, a total of 65 tumors were treated with MWA over 65 sessions. None of the patients received adjuvant chemotherapy after MWA treatment.

**Table 1:** Patient demographics and tumor characteristics (initial treatment)

Characteristic	Number of patients (%)
Gender	
Male	40 (63.5%)
Female	23 (36.5%)
Age (years)	82.1 ± 3.2
Smoking history	
Yes	25 (39.7%)
≥400	19 (30.2%)
Comorbidities	
COPD	33 (52.4%)
Coronary heart disease	33 (52.4%)
Diabetes	12 (19.0%)
Hypertension	34 (53.9%)
Cerebrovascular diseases	22 (34.9%)
Arrhythmia	1 (1.6%)
Silicosis	1 (1.6%)
Charlson Comorbidity Index	1.25 ± 1.00
Tumor size (cm)	2.66 ± 0.89 (1.1–5.0)
≤3.0	42 (64.6%)
3.0–5.0	23 (35.4%)
Pathology	
Adenocarcinoma	47/65 (72.3%)
Squamous cell carcinoma	17/65 (26.2%)
Large cell lung cancer	1/65 (1.5%)

COPD, chronic obstructive pulmonary disease.

### Efficacy of local tumor ablation

**Figure 1:**
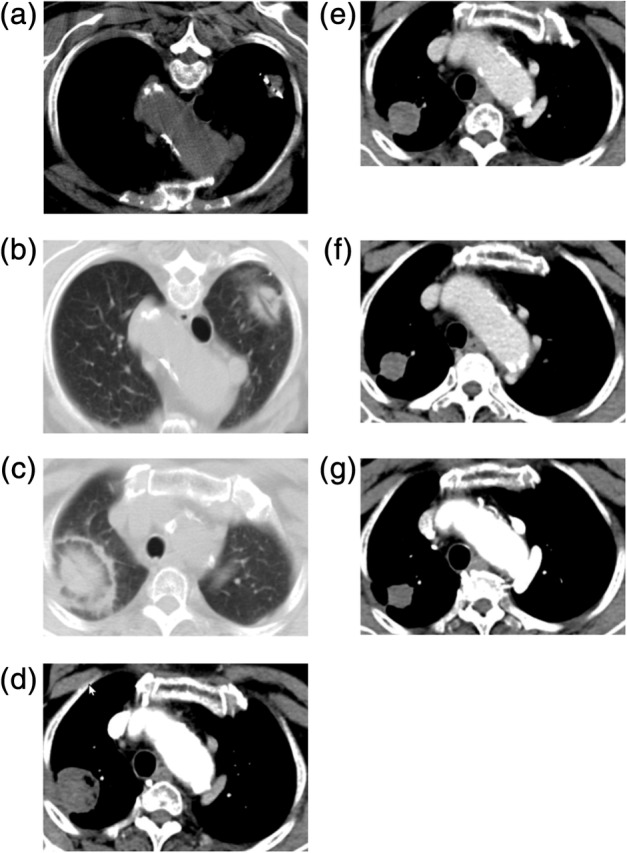
CT of an 80‐year‐old woman with right upper lobe adenocarcinoma at stage IA (cT1aN0M0). (**a**) The MWA antenna is shown centrally positioned within the tumor. (**b**) Ground‐glass opacity around the tumor and cavity in the core was observed in the image obtained immediately postablation. (**c**) The axial CT with lung window showed expected thermal damage around the target lesion, without pneumothorax, 24 hours postablation. (**d**) The enhanced CT image at one month after MWA; the size of the ablation zone was larger than the target tumor but was not enhanced; (**e**–**g**) The images at six, 12, and 19 months after MWA; the ablation zone shrank continuously and remained nonenhanced. CT, computed tomography; MWA, microwave ablation.

**Figure 2:**
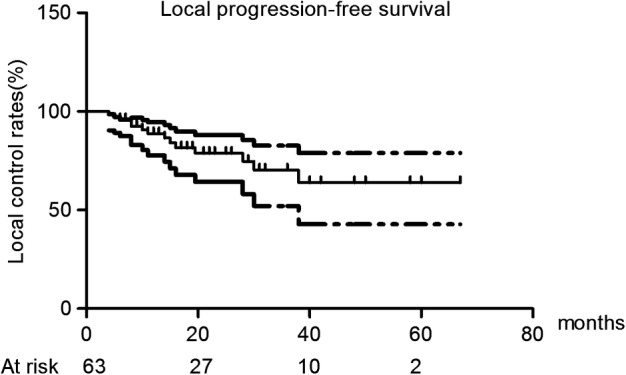
The Kaplan‐Meier plot of local control rates.

All initial treatment sessions were completed according to the planned protocol and deemed technically successful. The technique effectiveness rate was 100%. A total of 51 primary tumors (51/65, 78.5%) that were completely ablated remained stable until the end of follow‐up (Fig 1). Six patients with local recurrence underwent six sessions of secondary ablation, and none of them had secondary local recurrence by the end of follow‐up. The one‐, two‐, three‐, four‐, and five‐year local control rates (including the primary and secondary therapy sessions) were 88.8%, 78.8%, 70.3%, 63.9%, and 63.9%, respectively (Fig 2). One 80‐year‐old man with lung adenocarcinoma received two cycles of pemetrexed monotherapy after secondary ablation. One patient with local progression underwent radiotherapy but did not complete the treatment plan owing to physical intolerance to the therapy.

### Survival

The median postablation follow‐up time was 21.0 months (range: 6–70 months). Seven patients died from noncancer‐related causes, while nine died of tumor progression.

**Figure 3:**
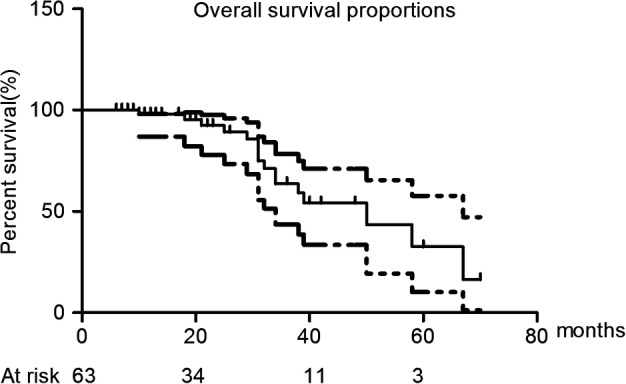
The Kaplan‐Meier plot of overall survival rate.

**Figure 4:**
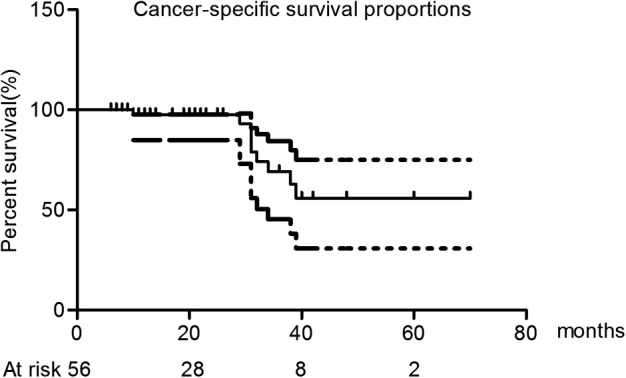
The Kaplan‐Meier plot of cancer‐specific survival.

The median OS was 50.0 months (95% confidence interval: 31.4–68.6 months). The estimated one‐, two‐, three‐, four‐, and five‐year OS rates were 97.1%, 92.6%, 63.4%, 54.4%, and 32.6%, respectively (Fig 3). The Kaplan‐Meier median cancer‐specific survival (CSS) was not reached by the end of follow‐up, and the one‐, two‐, and three‐year CSS rates were 97.9%, 97.9%, and 69.4%, respectively (Fig 4). The four‐ and five‐year CSS rates were not reached.

### Complications and side effects

The mortality rate was 0.0% within 30 days after MWA. The major complications in this study included pneumothorax requiring catheter drainage, pleural effusions requiring chest tube placement for drainage, and pulmonary infection. The overall incidence of pneumothorax requiring drainage was 21.1% (15 out of 71 primary and secondary sessions. Three patients with pneumothorax had coexisting subcutaneous emphysema. Two patients with hydrothorax required interventional drainage. Two patients developed bacterial pneumonia that was managed with sputum culture‐specific antibiotics. Two patients developed fever (temperature: >38.0°C), but no clear etiological evidence was obtained; the patients' symptoms were alleviated after treatment with empirical antibiotics. One patient with pulmonary cavitation at the ablation site developed pulmonary aspergillosis six months after the procedure that was treated and cured with voriconazole treatment. None of the patients had massive hemoptysis or thoracic hemorrhage requiring blood transfusion. The incidence of pleural effusions requiring drainage was 2.8% (2/71), and the incidence of *Aspergillus* infection was 1.59% (1/63).

In this study, none of the procedures were interrupted owing to complaints of pain. The patients developed the following side effects: pain, postablation syndrome, and asymptomatic minor bleeding or fluid accumulation on CT. Only three patients experienced mild pain at the puncture site for more than one month. The pain experienced by the other patients disappeared within one week. Five patients developed postablation syndrome characterized by fatigue, general discomfort, and anorexia. The symptoms disappeared after three days of treatment with dexamethasone. CT obtained 24 hours after each MWA session showed that almost all patients had pleural exudation on the affected side, and some also had contralateral pleural exudation that did not require puncture or catheterization.

## Discussion

The possibility that patients with early NSCLC will not receive any treatment increases with age.^15^ The survival of patients with stage I NSCLC who do not receive any form of treatment is not optimistic. A retrospective study based on the National Cancer Database of America analyzed the stage‐specific median and five‐year survival of untreated patients with NSCLC whose cancers were classified as operable and nonoperable.^16^ The study demonstrated that the median survival time of patients with stage I NSCLC who were operable but were untreated was 16.6 months, and the five‐year survival rate was 10.1%. The survival rate of untreated patients with contraindications for surgery was poorer: the median survival time and five‐year survival rate were 7.6 months and 4.0%, respectively. The survival rate of patients treated with MWA in our study was better than that of untreated patients in the retrospective study. Jeppesen *et al*. compared the clinical outcomes of patients with inoperable T1‐2N0M0 NSCLC treated with stereotactic body radiation therapy (SBRT) to untreated patients.^17^ The average ages of the two groups were 73 and 78 years; the mean OS from the date of diagnosis was 40 months and 9.9 months; and the five‐year survival rates were 37.0% and 6.0% for the treated and untreated groups, respectively. Another retrospective study reported that the median OS was 29 months in patients who underwent SBRT versus 10.1 months in those who were not treated.^18^ Despite the fact that the untreated patients were older than those treated with SBRT in the two studies mentioned above, the five‐year survival rate and median OS found in our study were comparable.

SBRT is the standard of care for medically inoperable patients with early‐stage NSCLC.^19^ However, whether the excellent local control rates of SBRT would result in an OS benefit remains unclear. The American College of Surgeons Oncology Group (ACOSOG) Z4033 study found that although the local control rate of the SBRT group was better than that of the RFA group, the two‐year overall survival rate of the RFA group was the same as that of the SBRT group, despite patients in the RFA group being older and having more underlying diseases than those in SBRT group.^20^ In 2010, Haasbeek *et al*. published a retrospective study of 193 patients with NSCLC aged over 75 years who received SBRT treatment.^21^ The median follow‐up time was 12.6 months, and the three‐year local control rate was 89%, which are better than those reported in our study (70.3%). Nevertheless, the good local control rate of SBRT in this study did not result in a better OS: the overall one‐ and three‐year survival rates were 86% and 45%, respectively, compared with 97.1% and 63.4%, respectively, in our study. In addition, some patients in our study who developed local progression received additional MWA therapy, reflecting the repeatability of the technique. In contrast, the risk of radiation pneumonitis and fibrosis, especially in patients with medically inoperable NSCLC and interstitial lung disease or other conditions that cause borderline lung function, makes it difficult to provide repeat SBRT. Moreover, the use of SBRT is limited and can only be administered in five daily treatments, which is more distressing to patients and their families than a single ablation procedure. In our study, an 80‐year‐old woman with squamous cell carcinoma who was diagnosed with local recurrence 30 months postablation failed to complete the radiotherapy plan owing to intolerance of the treatment.

Percutaneous pulmonary thermal ablation is a relatively safe and minimally invasive treatment. There are almost no absolute contraindications except for irreversible coagulation dysfunction or thrombocytopenia. In some medical institutions, the treatment is offered as an outpatient procedure. Patients with pretreatment pulmonary dysfunction may experience temporary exacerbation of respiratory symptoms after ablation and require oxygen therapy lasting one day to three weeks.^22^ It is difficult to clearly define the lowest threshold of pulmonary reserve that can tolerate percutaneous pulmonary thermal ablation. de Baere *et al*. reported that they successfully performed percutaneous lung RFA in a patient with a forced expiratory volume in one second (FEV1) of <0.8 L/s.^23^ In a previous study of patients who received lung RFA, three of 10 patients with FEV1 of <1 L developed transient respiratory insufficiency.^24^ The overall good tolerance of percutaneous pulmonary thermal ablation makes it possible to propose repeated treatments in patients with local recurrence or new metastases. In our study, there were 13 patients with COPD and one patient with silicosis. Two of these patients had FEV1 of <1 L/s, yet none of them developed respiratory failure within 30 days after the procedure. Dupuy *et al*. found that despite the thermal effects in the lung surrounding the tumors, there were no deleterious effects on pulmonary function.^20^ They also found that forced vital capacity was significantly improved after ablation compared to before treatment, which may be explained by a reduction in air trapping owing to the cicatrization effects of RFA. Moreover, Timmerman *et al*. found that the carbon monoxide diffuse capacity of patients decreased by 12% after SBRT.^25^ Together, these results suggest that percutaneous pulmonary ablation is more effective than SBRT in protecting lung function.

The 30‐day mortality rate in the current study was 0.0%, which was consistent with those reported in previous studies.^26^, ^27^, ^28^, ^29^, ^30^, ^31^, ^32^ The incidence of pneumothorax requiring drainage in our study was higher than that previously reported. This may be related to the presence of COPD in 52.4% of the patients in this study. Zheng *et al*. found emphysema as a risk factor for pneumothorax requiring catheter drainage after MWA.^33^ The incidence of pleural effusions requiring drainage in the current study is in accordance with that reported in previous studies,^20^, ^33^ and the incidence of *Aspergillus* infection in the current study is consistent with that reported by Huang *et al*. (1.44%).^34^

This study had several limitations: (i) it used a small sample size; (ii) it was retrospective in nature; (iii) data on factors that could influence outcomes were unavailable; and (iv) multiple devices were used by multiple operators.

In conclusion, MWA is a safe and effective treatment for patients aged 80 years and older with early‐stage peripheral lymph node‐negative NSCLC. The results of this study support the use of MWA as a treatment in older patients who may otherwise be left untreated, thereby leading to improved outcomes in these patients. Additional studies, including well‐designed and sufficiently powered randomized trials, are necessary to further confirm our results.

## Disclosure

The authors declare that they have no competing interests.
